# Lymphatic Connexins and Pannexins in Health and Disease

**DOI:** 10.3390/ijms22115734

**Published:** 2021-05-27

**Authors:** Avigail Ehrlich, Filippo Molica, Aurélie Hautefort, Brenda R. Kwak

**Affiliations:** Department of Pathology and Immunology, University of Geneva, CH-1211 Geneva, Switzerland; avigail.ehrlich@unige.ch (A.E.); filippo.molica@unige.ch (F.M.); aurelie.hautefort@unige.ch (A.H.)

**Keywords:** gap junction, connexin, pannexin, lymphatic vasculature, lymphedema

## Abstract

This review highlights current knowledge on the expression and function of connexins and pannexins, transmembrane channel proteins that play an important role in intercellular communication, in both the developing and mature lymphatic vasculature. A particular focus is given to the involvement of these proteins in functions of the healthy lymphatic system. We describe their influence on the maintenance of extracellular fluid homeostasis, immune cell trafficking to draining lymph nodes and dietary nutrient absorption by intestinal villi. Moreover, new insights into connexin mutations in primary and secondary lymphedema as well as on the implication of lymphatic connexins and pannexins in acquired cardiovascular diseases are discussed, allowing for a better understanding of the role of these proteins in pathologies linked to dysfunctions in the lymphatic system.

## 1. Introduction

Twenty one gap junction proteins, called connexins (Cxs), are found in humans, compared to twenty in mice [1,2]. Cx proteins are named after their specific molecular weight in kDa, whereas Cx genes follow a subfamily classification (*GJA*–*GJE*) with numbering according to their chronology of discovery. The topology of Cxs consists of four α-helical transmembrane (TM) domains, two extracellular loops (ELs), an intracellular loop (IL) and a cytosolic amino- and a carboxyl-terminal (NT and CT) domain. Cxs form hexamers, called connexons, in the endoplasmic reticulum or Golgi apparatus, which traffic thereafter to the cell membrane where they may function as hemi-channels participating in exchanges between the cell and its environment [3]. Gap junction channels are formed by the docking of connexons from two adjacent cells, allowing the diffusion of small molecules (< 1 kDa) and ions between their cytoplasms [4,5]. Another family of transmembrane proteins, called pannexins (Panxs), share the same topology as Cxs but with different sequences. This family of channel-forming proteins consists of only three members, namely Panx1–Panx3 [6]. One principal difference between Cxs and Panxs is the high level of glycosylation within Panx ELs, often referred to as “preventing gap junction formation”, which is inappropriate as Panx channels are not gap junction hemi-channels [7]. Reports of further essential differences between these protein families came from recent structural studies demonstrating that Panx channels (pannexons) result from the assembly of seven (rather than six) Panxs [8,9]. All three Panxs are expressed in humans and mice and share 94% sequence homology [6]. Cxs and Panxs are widely expressed in different cell types and tissues, including the lymphatic system [10,11,12]. In this review, we will focus on the role of the communication channels formed by Cxs and Panxs in the development and function of the lymphatic network. The effect of mutations in the sequences of these proteins in lymphatic pathologies will be discussed and the potential role of lymphatic Cxs and Panxs in acquired cardiovascular diseases will be described.

## 2. Functions of the Lymphatic System

**Figure 1:**
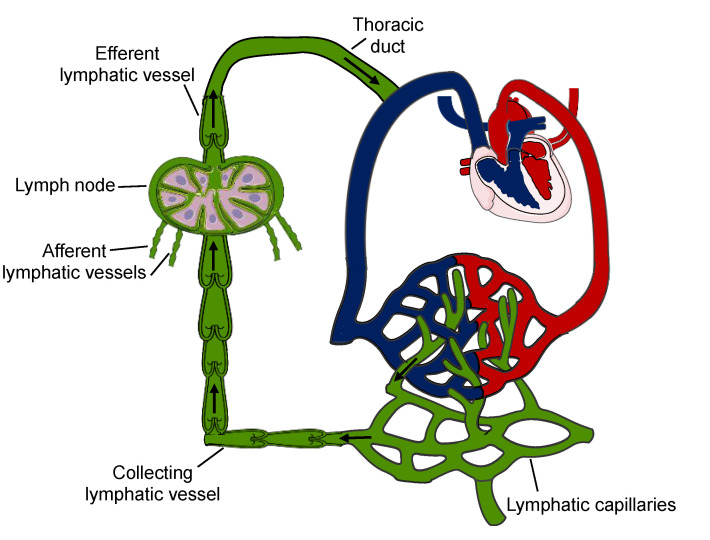
Unidirectional lymph transport. Lymphatic capillaries collect peripheral macromolecules, fluids and cells and then converge into larger collecting vessels containing lymphatic valves, which assures the unidirectional flow of the lymph. These afferent lymphatic vessels subsequently drain to lymph towards the lymph nodes. An efferent lymphatic vessel transports the lymph via the thoracic duct to the subclavian vein into the systemic circulation. Arrows indicate the direction of lymph flow.

The lymphatic system is a network of capillaries and collecting lymphatic vessels allowing for the unidirectional transport of interstitial fluids to the venous system [13]. It is present in nearly all vascularized tissues [14]. The lymphatic capillaries initiate in the interstitial space of organs and collect fluids, macromolecules and cells; they then converge into pre-collectors and collecting lymphatic vessels to drain the lymph towards lymph nodes. From here on, the lymph is transported into the thoracic duct or the right lymphatic trunk to finally join the blood circulation (Figure 1).

**Figure 2:**
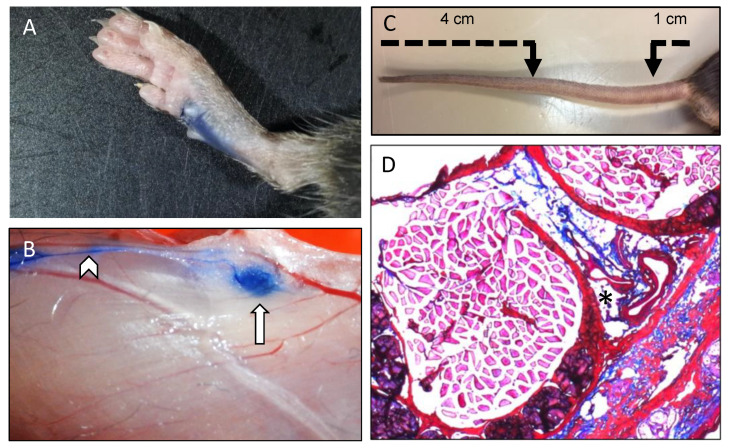
Methods to determine drainage of interstitial fluids. (**A**) Photograph shortly after injection of 5 microliters of 5% Evans blue in the footpad. (**B**) Representative image illustrating draining through lymphatic vessels (arrowhead) to a draining lymph node (arrow) at 15 min after the footpad injection with Evans blue. (**C**) The tail diameter is typically measured with a caliper at two different locations (1 cm from the basis and 4 cm from the tip of the tail) to determine swelling, indicative of the presence of edema. (**D**) Representative image of Masson’s trichrome staining on a tail cryosection illustrating the presence of edema in close proximity to blood and lymphatic vasculature (black asterisk).

Lymphatic vessels actively regulate tissue fluid homeostasis, trafficking of immune cells to draining lymph nodes (LNs) and absorption of dietary fats [15]. Under physiological conditions, the blood plasma is constantly filtered from the intravascular compartment, through the capillary walls into the interstitial space. Starling forces importantly regulate re-absorption of interstitial fluids towards the intravascular space at the venous side of capillary beds, and the lymphatic system transports the excess of this fluid (up to 2–4 L per day) back to the systemic circulation. The lymphatic system is thus a crucial contributor to tissue fluid homeostasis, and a typical manifestation of its dysfunction is lymphedema, a debilitating condition in which excess fluid accumulates in tissues and causes swelling [14]. In mice, quantification of the lymphatic drainage function is commonly performed using a defined injection of Evans blue in the footpad and measuring the amount of the dye accumulated in draining LNs or in the blood per unit of time (Figure 2A,B) [16,17]. Alternatively, tail swelling in mice results from interstitial fluid accumulation (edema) and measurement of tail diameters using a digital caliper provides information about dysfunctional lymphatic drainage [18,19] (Figure 2C). Edema can also be shown by Hematoxylin–Eosin (HE) or Masson’s trichrome staining on cryosections of tails (Figure 2D). A second crucial function of the lymphatic system is to act as a conduit for the trafficking of immune cells from the periphery to the LNs and to drain soluble antigens [14]. Indeed, tissue-resident dendritic cells (DCs) take up antigens and migrate to draining LNs for antigen presentation to start T cell responses [20]. Soluble antigens are also directed to draining LNs but faster than DCs and are thought to prime the LN for the arrival of antigen-presenting DCs [21]. It is important to mention that DCs do not passively follow lymphatic flow but they actively interact with lymphatic endothelial cells (LECs) to enter into lymphatic vessels and to exit the lymphatic vessel lumen towards the LN parenchyma, and that this process is critically guided by various chemokines and adhesion molecules [22]. A contact hypersensitivity assay is a common functional test to measure in vivo DC migration in mice [16,23]. In this assay, the percentage of migratory DCs arriving in draining LNs 24 h after exposure of the skin in the right flank to acetone/dibutyl phthalate is compared to the DCs found in non-draining contralateral LNs.

Another important function of the lymphatic system is to allow absorption of dietary nutrients from the gut, which depends critically on villi in the small intestine [14]. These small finger-like extensions from the epithelial cells covering the lumen of the small intestine contain a blood capillary network as well as blind-ended central lymphatic vessels called lacteals [24]. Short- and medium-chain fatty acids, carbohydrates and amino acids are taken up by blood capillaries in the villi, whereas lacteals absorb long-chain fatty acids and fat-soluble vitamins and transport these nutrients to the systemic circulation for distribution to all cells of the body. Lipid absorption functional tests in mice include the oral lipid tolerance test (OLTT). After a fasting period, mice receive a defined lipid bolus (olive oil) by oral gavage and serum concentrations of triglycerides and free fatty acids are determined at fixed time points thereafter [19,24]. Of further note, lacteals also play an essential role in the gut immune response.

## 3. Cx Expression in Lymphatic Vessels In Situ

**Figure 3:**
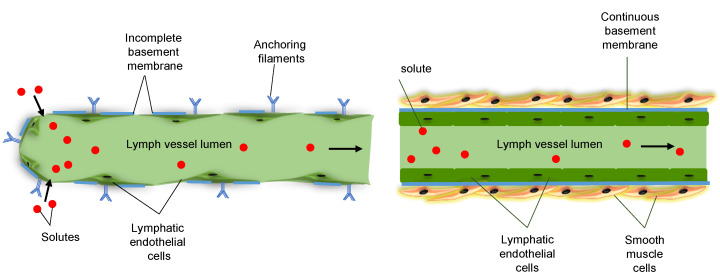
Structure of lymphatic vessels. (**left**) Lymphatic capillaries initiate blind-ended in the interstitial space of organs and tissues. They lack a basement membrane and have discontinuous intercellular junctions, which favors the uptake of interstitial fluid. LECs of lymphatic capillaries are attached by anchoring filaments to the extracellular matrix, which prevents vessel collapse under conditions of increased interstitial pressure. (**right**) Lymphatic collecting vessels have a continuous basement membrane and are surrounded by smooth muscle cells. The spontaneous phasic contractility of smooth muscle cells induces a coordinated propulsion of lymph.

LECs of lymphatic capillaries are attached by anchoring filaments to the extracellular matrix (ECM), which prevents vessel collapse under conditions of increased interstitial pressure. Lymphatic capillaries lack a basement membrane and have discontinuous intercellular junctions, favorable conditions for the uptake of interstitial fluid (Figure 3, left). Lymphatic capillaries drain via pre-collecting vessels into lymphatic collecting vessels, which have a continuous basement membrane and are surrounded by smooth muscle cells (SMCs) (Figure 3, right). Collecting lymphatic vessels are composed of lymphangions, a series of functional units that are separated by intraluminal valves, which ensure unidirectional lymph flow [12]. Lymphatic valves consist of two semilunar leaflets, covered on both sides by lymphatic endothelium. Opening and closure of lymphatic valves depend on periodic changes of fluid pressure gradients within the collecting vessels [25]. Lymph propulsion along the lymphatic network depends on intrinsic pump forces, generated by the spontaneous phasic contractility of SMCs that cover each lymphangion. Rhythmic compression and expansion of lymphatic vessels by surrounding tissues further ensures lymph flow. 

Since Compton and Raviola showed in 1985 by electron microscopy that LECs of the marginal sinus of the rabbit popliteal lymph node contain gap junctions [26], several studies focused on the expression of Cxs and the role of gap junctions in the lymphatic vasculature. An initial study reports the expression of Cx43 in the sinus-lining endothelial cells of LNs [27]. Functional studies in the mesentery of the rat small intestine showed that lymphatic vessels display coordinated contractions that were propagated both antegrade and retrograde to the direction of lymph flow, suggesting, in analogy to blood microvasculature, that gap junction communication may be responsible for the propagation of coordinated contraction. Indeed, the use of the non-specific gap junction inhibitors heptanol or oleic acid in experiments on isolated lymphatic vessels supported this hypothesis [28].

More recently, three Cxs have been consistently found in LECs of collecting lymphatic vessels, i.e., Cx37, Cx43 and Cx47 [16,29,30]. Cx37 and Cx43 are already observed at an early developmental stage in LECs of the jugular lymph sac but are also found in LECs of adult lymphatic vessels and lymphatic valves. While the two Cxs colocalize in the LECs of lymphangions, their expression pattern is highly segregated in LECs covering lymphatic valves; Cx43 is abundant in the upstream leaflets, whereas Cx37 is enriched in LECs of downstream leaflets. In addition, Cx47 is expressed in a small subset of LECs covering the upstream side of the adult lymphatic valve (in co-expression with Cx43) [29]. In a recent study re-assessing the coordination of lymphatic propulsive contractions, Castorena-Gonzalez and colleagues identified Cx45 as being the major Cx family member expressed in lymphatic SMCs in murine popliteal lymphatic vessels and in human mesenteric lymphatic vessels [31]. Finally, ablation of ectodermal Cx26 in mice resulted in embryonic death with extended lymphedema [32]. In an earlier study, Cx26 expression in human breast tumors was associated with lymphatic vessel invasion [33]. Although these findings suggest an involvement of ectodermal/epithelial Cx26 in the signaling regulating lymphangiogenesis, it should be kept in mind that expression of Cx26 in the LECs themselves has never been demonstrated.

## 4. Cx and Panx in Lymphatic Development

Cxs play an essential role in the homeostasis of blood and lymphatic vessels [12]. Cxs and gap junctions are also important for the development and the function of the lymphatic vasculature [29]. Cx37 and Cx43 are highly expressed during development of the lymphatic system. Early in development, these Cxs are typically expressed in the jugular lymph sacs, and later in development these Cxs become enriched in the lymphatic valve regions with the typical non-overlapping expression pattern in LECs on the upstream and downstream sides of the valves [29]. Compared with wild-type (WT) mice, jugular lymph sacs are enlarged at E13.5 in *Cx37^−/−^* and *Cx37^−/−^Cx43^−/−^* mice but not in *Cx43^−/−^* mice. Furthermore, *Cx37^−/−^Cx43^−/−^* mice exhibit abnormal thoracic duct development, defective lymphatic valve formation and lymphedema at E18.5 and at birth [29]. Markedly, *Cx37^−/−^Cx43^+/−^* mice had a chylothorax at birth and exhibited reduced postnatal viability. Chylothorax was not observed in *Cx43^+/−^* mice and was extremely rare in *Cx37^−/−^* mice. As signs of retrograde lymph flow were also observed in *Cx37^−/−^Cx43^+/−^* mice, the authors proposed that the chylothorax may be due to a failure to move lymph in an unidirectional (anterograde) manner [29]. Finally, postnatal LEC-specific deletion of Cx43 in *Cx37^−/−^* mice resulted in rapid regression of valve leaflets and severe valve dysfunction [34].

The formation of lymphatic valves is initiated around E16 and involves a four step process [30]. The first signs of embryonic lymphatic valve formation involve an upregulation of prospero homeobox 1 (Prox1) shortly thereafter followed by an upregulation of forkhead box C2 (Foxc2) in the LECs of the future valve region. Clusters of such lymphatic valve-forming cells coalesce and form a ring-like constriction. The highly coordinated formation of these ring-like constrictions suggested that direct cell-to-cell communication mediated by gap junctions may regulate this process. Although blood vessel formation in *Cx37^−/−^* mice appeared normal, most lymphatic vessels in *Cx37^−/−^* mice displayed a severely reduced number of lymphatic valves, resulting in perturbed lymphatic drainage in embryos and adult mice [11,30,35]. Mechanistically, an important contribution of mechanical forces to the valve remodeling process was demonstrated. In fact, oscillatory shear stress regulated Cx37 expression, activation of calcineurin/nuclear factor of activated T-cells and cytoplasmic 1(NFATc1) signaling, a response that required both Prox1 and Foxc2. Subsequently, Cx37 and calcineurin appeared to control distinct steps of lymphatic valve formation in vivo [30]. Interestingly, mutations in Foxc2 result in lymphedema-distichiasis syndrome in humans [36,37]. Kanady and colleagues generated *Foxc2^+/−^Cx37^−/−^* mice and observed that these mice typically die perinatally and suffer from generalized lymphedema in utero, craniofacial abnormalities, severe dilation of intestinal lymphatics, abnormal lacteal development and lack of lymphatic valves [38]. The worsening of lymphatic defects in *Foxc2^+/−^Cx37^−/−^* mice further confirmed that Foxc2 and Cx37 are elements in a common molecular pathway directing lymphangiogenesis. Mechanical forces, including shear stress and matrix stiffness, are increasingly recognized as key regulators of cell behavior and fate during development, homeostasis and disease. It is therefore of particular interest that culturing LECs on a soft matrix of 0.2 kPa, which mimics the stiffness of embryonic tissue allowing LEC progenitor migration, led to GATA-binding factor 2 (GATA2)-dependent upregulation of many genes controlling valve morphogenesis, including Cx37 [39].

Ubiquitous knock-out of Cx43 in mouse embryos leads to complete lack of mesenteric lymphatic valves at E18.5 [29]. As *Cx43^−/−^* mice die perinatally due to cardiac outflow tract malformation [40], the possibility that the lack of valves may be attributed to the deletion of Cx43 in other cells than LECs could not be excluded. Recently, this concern was addressed by the generation of mice with a lymphatic-specific ablation of Cx43 [41]. The absence of Cx43 in LECs caused a delay rather than a complete block in the initiation of lymphatic valves, leading to an increase in immature valves with incomplete leaflet elongation and a reduction in the total number of valves. Altered lymphatic capillary patterning was also observed. Although these mice reach adulthood, the consequences of these developmental defects are severe, i.e., leaky valves, insufficient lymph transport and reflux and a high incidence of lethal chylothorax [41]. Of note, Cx37 levels were not affected by the absence of Cx43; however, Cx47 expression was strongly decreased in thoracic duct valves compared to control valves [41].

Although mutations in gap junction protein gamma-2 (*GJC2*), the gene encoding Cx47, were the first shown to be implicated in lymphedema, Cx47 was only observed in a subset of LECs at upstream sides of lymphatic valves of embryonic mice [29]. The expression and the function of Cx47 in lymphatic vessels of adult mice was investigated by Meens and colleagues [16]. They showed that Cx47 is expressed in adult lymphatic endothelium, but it seemed only modestly implicated in lymphatic pathophysiology. Indeed, lymphatic contractility, lymphatic morphology, interstitial fluid drainage or DC migration through lymphatic vessels was not affected in *Cx47^−/−^* mice. Although Cx47 appeared also dispensable for long-chain fatty acid absorption from the gut, it seemed to promote serum lipid handling as prolonged elevated triglyceride levels were observed in *Cx47^−/−^* mice after OLTT [16].

As described above, Cx45 expression was shown in human and mouse lymphatic smooth muscle [31], where it mediates SMC–SMC electrical coupling and thus the conduction of pacemaker signals and entrained contractions. As such, smooth muscle-specific ablation of Cx45 in mice resulted in reduced electrical conduction, loss of contractile coordination and impaired lymph pump function ex vivo. Under an imposed gravitational load (near-vertical body position), these mice showed inhibition of in vivo lymph transport, which could not be observed in a horizontal position. Interestingly, lymphatic vessels from mice with smooth muscle-specific ablation of Cx45 displayed a higher number of pacemaking sites, but none carried a dominant role, leading to inefficient contractions and reduced ejection fractions from their lymphangions [34]. This pattern further worsened in these lymphatic vessels when they were exposed to elevated downstream pressure. The authors concluded that efficient lymph transport not only needs strong entrained contractions of all SMCs across multiple lymphangions, but also requires a dominant pacemaker for which Cx45-mediated electrical coupling between lymphatic SMCs is critical [34].

As a key player in intercellular communication, Panx channels may represent a potential new actor in the regulation of lymphatic function. In this context, Panx1 mRNA expression was found in LECs in mice [19]. Interestingly, Panx1-deficient mice displayed impaired lymphatic function with reduced dietary fat absorption and decreased drainage of interstitial fluids [19]. More recently, a role for Panx1 in in vitro lymphangiogenesis was described [42]. Using primary human dermal lymphatic cells, the authors first showed that vascular endothelial growth factor C (VEGF-C), the principal regulator of lymphangiogenesis, increased Panx1 expression. Moreover, chemical inhibition of Panx1 channels or siRNA knockdown of Panx1 reduced capillary tube formation in Matrigel, which appeared due to an impaired invasion response to VEGF-C rather than due to decreased cellular proliferation [42]. Altogether, these studies will most likely lead to investigations towards the contribution of Panx1 in lymphatic vessel development.

## 5. Cx Mutations and Lymphedema

Lymphedema is characterized by an accumulation of extracellular fluid in the interstitial spaces of limbs due to developmental or functional lymphatic anomalies [43]. Lymphedema can be of primary or secondary origin. Although highly heterogenous in onset, primary lymphedema implies a genetic cause. Secondary lymphedema is acquired and appears as a result of an underlying infection, trauma or surgery [44]. Mutations in a variety of genes regulating the development of lymphatic vessels, including Cxs, may result in inherited lymphedema [36,43].

Missense mutations in *GJC2* leading to substitutions in conserved amino acids in Cx47 have been shown to be implicated in the development of autosomal dominant late-onset four-limb lymphedema [45,46]. Although these patients showed inter-familial variability in clinical severity, lymphoscintigraphy pointed to reduced absorption of isotopes from the tissue by the peripheral lymphatics, suggesting a problem in the absorption of fluid by lymphatic capillaries [46]. Some of the patients also suffered from varicose veins, suggestive of additional venous valve failure [46]. Interestingly, a later study revealed by ultrasound imaging that individuals with *GJC2* mutations possessed fewer venous valves with shorter leaflets [47]. *GJC2* mutations have also been described in patients suffering from Pelizaeus-Merzbacher-like disease (PMLD), a hypomyelinating leukoencephalopathy, in which lymphedema does not occur [36]. Mutations in PMLD are frequently of the “loss-of-function” type, involving premature stop codons and mutations in the *GJC2* promoter, and generally segregate from the mutations found in lymphedema patients with only one exception for G146S [48]. Before describing the lymphedema-linked *GJC2* mutations in detail, it should be noted that the literature regarding Cx47 mutations has been somewhat confusing as amino acid sequences were originally based on the first ATG start site for human *GJC2*. Over the years, increasing evidence pointed to the second ATG site for initiation of translation for human *GJC2* and a progressive change was made by authors to using this site as a basis for numbering the amino acid sequence. We will use a nomenclature based on the second ATG site throughout this review, resulting in some of the amino acid substitutions in this review being numbered three amino acids less than in their original publications.

The reported mutations for primary lymphedema are missense mutations and result in amino acid substitutions in various domains of the Cx47 protein, i.e., NT (H16P), ELs (S45L and R257C), IL (R122Q and G146S) and CT (P313L, P381S and H409Y) [45,46,49] (Table 1). Interestingly, the S45L and R257C mutations are predicted to involve the EL regions and may interfere with the docking of connexons, thereby regulating gap junction formation [50,51].

The cellular and molecular consequences of the Cx47-G146S mutation have been studied after transfection in communication-incompetent cell lines. Gap junction plaques were detected at the plasma membrane in mutant-expressing cells. The mutated protein appeared enriched in the endoplasmic reticulum as compared to cells transfected with the WT Cx47. Next, resting membrane potentials and non-junctional transmembrane currents were studied in single *Xenopus laevis* oocytes using a two-electrode voltage clamp. Resting potentials of oocytes expressing WT and mutant Cx47 were not different; however, transmembrane currents observed for Cx47-G146S mutants were reduced, suggesting a loss-of-function in the mutant hemi-channels [52]. Unfortunately, the functional effects of the G146S mutation on gap junction channels was not assessed in this study.

Cx47 mutations have also been associated with an increased risk for secondary lymphedema following breast cancer treatment. In a case-control study Finegold and colleagues sequenced 81 breast cancer patients with secondary lymphedema and 108 control breast cancer patients for mutations in *GJC2* [53]. Four heterozygous missense mutations in different intracellular domains of Cx47, i.e., IL (G146S and G183C) and CT (P381S and H409Y), were found in the patient group with secondary lymphedema (Table 1). Of note, none of these patients reported a familial history of primary lymphedema, even though one of these patients had the same Cx47 mutation (G146S) as a family with primary lymphedema reported earlier by this research group [45]. Two other mutations (P381S and H409Y) have also been reported by Michelini and colleagues for patients with primary lymphedema [49] and one mutation is unique (G183C). Altogether, these data suggest that the breast cancer treatment may have exposed an up-till-then unrecognized primary lymphedema in these patients [53]. After expression in communication-incompetent HeLa cells or in LECs, some of the four mutations found in patients with secondary lymphedema showed a different functional phenotype from that found in cells expressing WT Cx47. Briefly, LECs expressing the H409Y mutation showed impaired transfer of Lucifer yellow after microinjection. In contrast, the P381S mutation caused enhanced dye transfer. Furthermore, G183C-transfected HeLa cells showed increased electrical coupling when compared with HeLa cells transfected with WT Cx47, whereas electrical coupling was not different in HeLa cells transfected with the mutations located in the CT domain (P381S and H409Y). Although these in vitro assays strongly suggest that lymphedema-related mutations may cause altered gap junction channel function, lots of work remains to be done before the mutations–gap junction function–lymphedema relationship is completely understood. As described above, lymphatic function is not dramatically altered in *Cx47^−/−^* mice [16,54], showing that deletion of Cx47 channels is not representative for the mutations found in humans. It is therefore possible that some heterozygous *GJC2* missense mutations lead to dominant negative effects on WT Cx47 or on other Cx channels, which may then be detrimental for gap junction communication and homeostasis in LECs. The creation of transgenic animals carrying specific missense mutations would be of tremendous help to resolve these ongoing questions.

Following the studies describing impaired lymphatic drainage in *Cx37^−/−^* mice, recently the hypothesis was tested that mutations or polymorphisms in gap junction protein alpha 4 (*GJA4*), the gene encoding Cx37, are associated with secondary lymphedema following breast cancer surgery [55]. A total of 2211 breast cancer patients were screened and tested for two single nucleotide polymorphisms (SNPs) in the 3’-UTR of the *GJA4* gene (rs3543 and rs705193), which is associated with an increased risk of secondary lymphedema in these patients. As the 3′-UTR of genes is often regulating RNA stability, the authors hypothesized that these SNPs may reduce the amount of Cx37 RNA resulting in decreased expression of the protein. Although this molecular mechanism was not further investigated, the authors proposed the use of these SNPs as novel genetic biomarkers for assessing the predisposition to secondary lymphedema in human breast cancer patients.

Oculodentodigital dysplasia (ODDD) is an inherited multisystem developmental disorder caused by around 80 distinct mutations in the *GJA1* gene encoding for Cx43 [56,57,58]. The disease is clinically characterized by soft tissue fusion of the fingers and toes, abnormal craniofacial bone development, small eyes and dental anomalies related to loss of tooth enamel. Patients variably present a number of additional co-morbidities involving a plethora of other organs including the central nervous system, skin and bladder. In 2013, a first case report described a patient with the characteristic ODDD symptoms and a bilateral lower limb lymphedema [59]. Lymphoscintigraphy confirmed a delay in drainage and the appearance was consistent with impaired lymphatic drainage in both legs. Genetic testing revealed a novel *GJA1* missense mutation leading to an amino acid substitution (K206R) in EL2 of Cx43 (Table 2). Two other family members with the same mutation presented with lymphedema, which is suggestive for causality. The K206R mutation involves the SRPTEK motif, a crucial region in the EL2 important for the docking of connexons and thus gap junction channel formation [60]. A second case of lymphedema in an ODDD patient related to a *GJA1* mutation was reported in 2018. Genetic testing revealed a heterozygous missense mutation leading to an amino acid substitution (G22Q) in TM1 of Cx43 (Table 2). On the basis of clinical evaluation and in silico analysis (PolyPhen, Mutation Taster) the variant was identified as pathogenic. Similar to other heterozygous mutations linked to ODDD, it is expected that the mutated copy exerts a dominant-negative effect on the normal allele, thereby likely contributing toward reduced Cx43-dependent gap junction communication.

## 6. Lymphatic Cxs and Panxs in Acquired Cardiovascular Diseases

Cxs and Panxs have been shown to be involved in different disease and repair processes with links to the lymphatic system, such as atherosclerosis and cardiac edema after myocardial infarction (MI), for instance.

The role of Cxs in atherosclerosis has been extensively studied for many years, while a potential involvement of Panx1 has only recently been highlighted [2]. Atherosclerosis is the principal cause of death worldwide [61]. It is a progressive disease of medium-sized and large arteries characterized by a dysfunctional endothelium, an accumulation of lipids and an abnormal inflammatory response leading to the formation of atherosclerotic plaques, and is linked to major cardiovascular outcomes such as MI and stroke [62]. It has been shown in atherosclerosis-susceptible mice that lymphatic vessel insufficiency affects lipoprotein levels and promotes atherosclerosis. Thus, atherogenesis, lymphatic function and lipoprotein metabolism are closely intertwined. In addition, lymphatic vessels may participate in atherosclerotic plaque regression by providing macrophages a way to exit from atherosclerotic plaques, this way promoting reverse cholesterol transport. As such, the presence of lymphatic vasculature in atherosclerotic lesions is considered as anti-atherogenic [63,64]. Cxs show altered expression patterns during atherogenesis. Cx43 is known as being atherogenic and reducing Cx43 decreases atherosclerotic plaque burden and enhances plaque stability [65,66]. Cx37, on the other hand, displays anti-atherogenic properties by reducing inflammatory cell recruitment and adhesion [67]. The lymphatic-specific role of Cxs has been less extensively studied. First, a potential role for lymphatic Cx47 in atherosclerosis was investigated using atherosclerosis-susceptible Apolipoprotein E-deficient (*Apoe^−/−^*) mice. As described above, Cx47-deficient mice had prolonged elevated triglyceride levels after OLTT [16]. Moreover, LDL-cholesterol levels in serum of old *Cx47^−/−^Apoe^−/−^* mice was increased as compared to *Apoe^−/−^* mice. In agreement with these altered serum lipids, advanced atherosclerotic lesions tended to be larger in old *Cx47^−/−^Apoe^−/−^* mice [16]. Secondly, a role for lymphatic Panx1 in atherosclerosis was dissected using two different mouse models. On the one hand, atherosclerosis-susceptible mice with a specific deletion of Panx1 in endothelial and monocytic cells (*Tie2-Cre^Tg^ Panx1^fl/fl^ Apoe^−/−^*) displayed increased atherogenesis, pointing to a protective role for Panx1 in these cells. On the other hand, atherogenesis was not changed in mice with ubiquitous Panx1 deletion, but *Panx1 ^−/−^Apoe^−/−^* mice had reduced body weight, serum cholesterol, triglycerides and free fatty acids, suggesting altered lipid metabolism in these mice [19]. Thus, a link between decreased lipid levels, atherosclerosis and a possible impairment in lymphatic function was hypothesized and lymphatic function tests performed. First, OLTT revealed reduced dietary fat absorption in *Panx1^−/−^Apoe^−/−^* mice after gavage with an olive oil bolus. Secondly, injection of Evans blue in the footpad of *Panx1^−/−^Apoe^−/−^* mice showed decreased drainage of interstitial fluids in *Panx1^−/−^Apoe^−/−^* mice, which was further confirmed by an increased tail diameter in these mice. Finally, an increased content of CD68-positive cells was observed in atherosclerotic plaques of *Panx1^−/−^Apoe^−/−^* mice as compared to *Apoe^−/−^* mice, which may be due to a lower efflux of these cells from the atherosclerotic plaques via lymphatic vessels. Taking all results together, the authors concluded that the detrimental effect of Panx1 deletion in endothelial and monocytic cells during atherogenesis was counterbalanced by an opposite effect resulting from impaired lymphatic function in ubiquitous Panx1-deficient mice [19]. Although these findings strongly suggest a link between Panx1, lipid metabolism, lymphatic function and atherosclerosis, a direct role for lymphatic Panx1 in this disease has not been demonstrated yet.

An important complication of atherosclerosis is MI. The heart has a unique system of lymphatic vessels covering the epicardial surface, which is most dense at the level of the ventricles [68]. In the adult heart, MI promotes a lymphangiogenic response, which reduces myocardial edema and fibrosis, leading to improved cardiac function and prolonged survival [69,70]. In an elegant study, Trincot and colleagues recently showed that adrenomedullin, an endogenous epicardial-derived factor, improves post-MI cardiac lymphangiogenesis and lymphatic function via effects of Cx43 [71]. They suggested that targeting Cx43 function may represent a new therapeutic pathway for improving myocardial edema after injury. In this respect, it is interesting to note that diverse Cx43-targeting strategies are underway for clinical testing. Indeed, a 30-mer antisense oligodeoxynucleotide (named Nexagon), which knocks down Cx43 expression, has undergone Phase II clinical testing for skin wounds [72]. Likewise, a 25-amino acid peptide that mimics the last nine amino acids of Cx43 (called aCT1) has been used in clinical trials in patients with chronic diabetic foot and venous leg ulcers [73,74].

## 7. Conclusions

Cx-built gap junction channels have been considered for over 50 years as integral components for cell-to-cell coupling within the cardiovascular system, and Panx channels have been more recently added to this repertoire of proteins for intercellular communication. While studies on Cxs and Panxs in the lymphatic system are at an infant stage, it is clear that these structures play an essential role in the normal functioning of this system in humans. Indeed, mutations in the *GJC2* and *GJA1* genes, encoding for Cx47 and Cx43, respectively, have been linked to primary and secondary lymphedema in various families and populations, which is of major importance for the stratification of lymphedema patients. Although the symptoms of human disease cannot be directly recapitulated in knockout mouse models, the development and function of the lymphatic system is severely perturbed in these mice. Still, it shows that the effects of specific gene mutations are likely more complex than a straightforward loss or gain of function. Whether we will be able to target Cxs for the treatment of lymphatic disorders will very much depend on the unraveling of the effects of the *GJC2* and *GJA1* gene mutations (and maybe in the future also *GJA4* and *Panx1* gene mutations) in relevant in vitro, ex vivo and in vivo models. In this respect, it is important to keep in mind that mounting evidence suggests that Cxs, and in particular Cx43, also have non-canonical channel-independent functions, including the modulation of cell differentiation, adhesion and gene transcription, which are believed to depend largely on their interacting partners. As protein–protein interactions may also be influenced by missense mutations leading to amino acid substitutions, we still have a long and exciting way to go in understanding all aspects of Cxs in lymphatic disease.

## Figures and Tables

**Table 1:** Overview of *GJC2* gene mutations found in primary and/or secondary lymphedema.

Amino Acid Substitution	Cx47 Domain	Functional Effect
H16P	NT	unknown
S45L	EL1	altered docking ^1^
R122Q	IL	unknown
G146S	IL	loss-of-function ^2^
G183C	IL	enhanced electrical coupling
R257C	EL2	altered docking ^1^
P313L	CT	unknown
P381S	CT	enhanced dye coupling
H409Y	CT	impaired dye coupling

^1^ prediction; ^2^ measured for Cx47-G146S hemi-channels.

**Table 2:** Overview of *GJA1* gene mutations associated with secondary lymphedema.

Amino ACID Substitution	Cx47 Domain	Functional Effect
Q22G	TM1	unknown
K206R	EL2	altered docking ^1^

^1^ prediction.
